# Comparison of multiple single-nucleotide variant association tests in a meta-analysis of Genetic Analysis Workshop 19 family and unrelated data

**DOI:** 10.1186/s12919-016-0028-7

**Published:** 2016-10-18

**Authors:** Shuai Wang, Virginia A. Fisher, Yuning Chen, Josée Dupuis

**Affiliations:** Department of Biostatistics, Boston University School of Public Health, Boston, MA 02118 USA

## Abstract

**Background:**

Meta-analysis has been widely used in genetic association studies to increase sample size and to improve power, both in the context of single-variant analysis, as well as for gene-based tests. Meta-analysis approaches for haplotype analysis have not been extensively developed and used, and have not been compared with other ways of jointly analysing multiple genetic variants.

**Methods:**

We propose a novel meta-analysis approach for a gene-based haplotype association test, and compare it with an existing meta-analysis approach of the sequence kernel association test (SKAT), using the unrelated samples and family samples of the Genetic Analysis Workshop 19 data sets. We performed association tests with diastolic blood pressure and restricted our analyses to all variants in exonic regions on all odd chromosomes.

**Results:**

Meta-analysis of haplotype results and SKAT identified different genes. The most significantly associated gene identified by SKAT was the *ALCAM* gene on chromosome 3 with a *p* value of 7.0 × 10^− 5^. Two of the most associated genes identified by the haplotype method were *FPGT* (*p* = 6.7 × 10^− 8^) on chromosome 1 and *SPARC* (*p* = 3.3 × 10^− 7^) on chromosome 5. Both genes were previously implicated in blood pressure regulation and hypertension.

**Conclusion:**

We compared two meta-analysis approaches to jointly analyze multiple variants: SKAT and haplotype tests. The difference in observed results may be because the haplotype method considered all observed haplotypes, whereas SKAT weighted variants inversely to their minor allele frequency, masking the effects of common variants. The two approaches identified different top genes, and appear to be complementary.

## Background

In recent years, genome-wide association studies (GWAS) have unearthed a large number of single-nucleotide variants (SNVs) associated with many diseases [1]. Most associated variants have been common, with a minor allele frequency (MAF) of greater than 5 % and have been identified using a 1-SNV-at-a-time approach, where association with each SNV is evaluated separately, ignoring linkage disequilibrium (LD) between SNVs. A large number of associated variants were discovered only after combining GWAS results from multiple cohorts using meta-analysis approaches. To better focus on rare variants, methods to jointly analyse variants have been developed with meta-analysis extensions to combine results from multiple cohorts [2, 3]. These methods include burden tests, where association between a trait and the number of rare alleles a person carries is evaluated, and the sequence kernel association test (SKAT), which aggregates the evidence for association over multiple SNVs allowing for different direction of effects [4]. All of these approaches ignore the possibility of haplotype effects, where multiple alleles inherited together influence the trait. Even though single cohort approaches for haplotype analysis have been developed [5, 6], meta-analysis of haplotype results remains challenging because of the possibility of cohort-specific haplotype structure.

In this paper, we compare two multi-SNV analysis approaches applied to the Genetic Analysis Workshop 19 (GAW19) data, using both family and unrelated data. We use meta-analysis to combine the results from SKAT and haplotype analysis, using a novel approach for meta-analysis of haplotype results developed by our group. We apply these approaches to subsets of SNVs defined by gene location.

## Methods

For all analyses considered, SNVs are grouped by gene location, and variants within each gene are tested for association with the phenotype of interest. Gene-based groupings were identified from the hg19 reference genome with the ANNOVAR (Annotate Variation) software [7]. From the family data set, we analyzed 464 individuals with sequence data available and 407 individuals from the unrelated data set.

We performed joint analysis of rare variants to assess association with diastolic blood pressure (DBP) adjusted for baseline age and sex. We adjusted DBP values for the use of blood pressure–lowering medication by adding ten to the observed DBP for all subjects reported to be on medication [8].

SKAT analyses were conducted with the RAREMETAL software [3]. Genome-wide single-variant association tests are calculated for family and unrelated samples separately with the RAREMETALWORKER software. The results were combined to calculate fixed-effect meta-analytic tests of association between the phenotype and groups of variants. The haplotype association test was implemented in R. Rare haplotypes (<0.5 %) were collapsed to ensure computational stability. To evaluate type 1 error rate of the haplotype analysis method, we analyzed genes on chromosome 17 from all 200 simulation replicates. We excluded genes located within 1 Mb of SNVs simulated to have an effect on any of the blood pressure traits.

### Sequence kernel association test

The SKAT method [4] is based on a regression model of phenotype as a function of covariates and genotypes at all loci within a region. Familial relatedness was incorporated at the cohort level by means of the expected kinship matrix calculated from the pedigree structure. For a gene-based group containing *p* variants, with effect parameters *β*_1_, ⋯, *β*_*p*_, the genotype-phenotype association is evaluated with the null hypothesis H_0_: *β*_1_ = *β*_2_ = ⋯ = *β*_*p*_ = 0. Specifically, under the assumption that each *β*_*j*_ is distributed with mean zero and variance *w*_*j*_ *τ* where *w*_*j*_ is a specified weight for SNV *j*, this is equivalent to the null hypothesis *τ* = 0, which is assessed by means of a variance components score test. In accordance with Wu et al. [4], weights are of the form *w*_*j*_ = *Beta*(*MAF*_*j*_; 1, 25)^2^ where the *Beta* distribution is evaluated at the MAF of that variant. This weights the rarest variants most heavily and smoothly reduces the weights of common variants, reflecting the assumption that natural selection against strong causal SNVs will result in lower frequency in the population. Several authors have developed approaches for meta-analysis of SKAT results, enabling the combination of GAW19 related and unrelated samples [2, 3].

### Haplotype association test

We developed a novel approach to test the association between haplotype structure and phenotype so as to better understand the genetic architecture of each region and its influence on DBP. We incorporated family structure into the model proposed by Zaykin et al. [6] so that our approach is applicable to both unrelated and related samples. For *K* observed haplotypes, we model the phenotype at the cohort level as

$$ \mathrm{Y}=\mathrm{X}\upgamma +{\beta}_1{h}_1+\dots +{\beta}_K{h}_K+b+\upvarepsilon $$

where *Y* is the trait (DBP at baseline), *X* are covariates such as age and sex with no intercept, *h*_*k*_ is the dosage of the *k* ^*th*^ haplotype out of *K* observed haplotypes, *b* ~ *N*(0, 2*σ* ^2^Σ_*kin*_) is a random effect vector that accounts for the familial correlation, Σ_*kin*_ is the expected kinship matrix derived from the pedigree and ε is the residual error. In our implementation, haplotype dosages are estimated from genotypes using an expectation–maximization algorithm (R Package haplo.stats [5]) ignoring familial information but exploiting the LD among genetic variants. We use a weighted least-squares method [9] to meta-analyze the beta coefficients for the K′ haplotypes observed in one or more cohorts. After meta-analysis, we test the global null hypothesis that all haplotype effects are equal (ie, H_0_: *β*_1_ = ⋯ = *β*_*K* '_). A Wald test with *df = K′ − 1* is implemented to test the reparameterized null hypothesis H_0_: *γ*_2_ = ⋯ = *γ*_K '_ = 0 where *γ*_*i*_ = *β*_*i*_ − *β*_*c*_ and the subscript “C” refers to one of the haplotypes common to all studies selected at random.

## Results

**Fig. 1:**
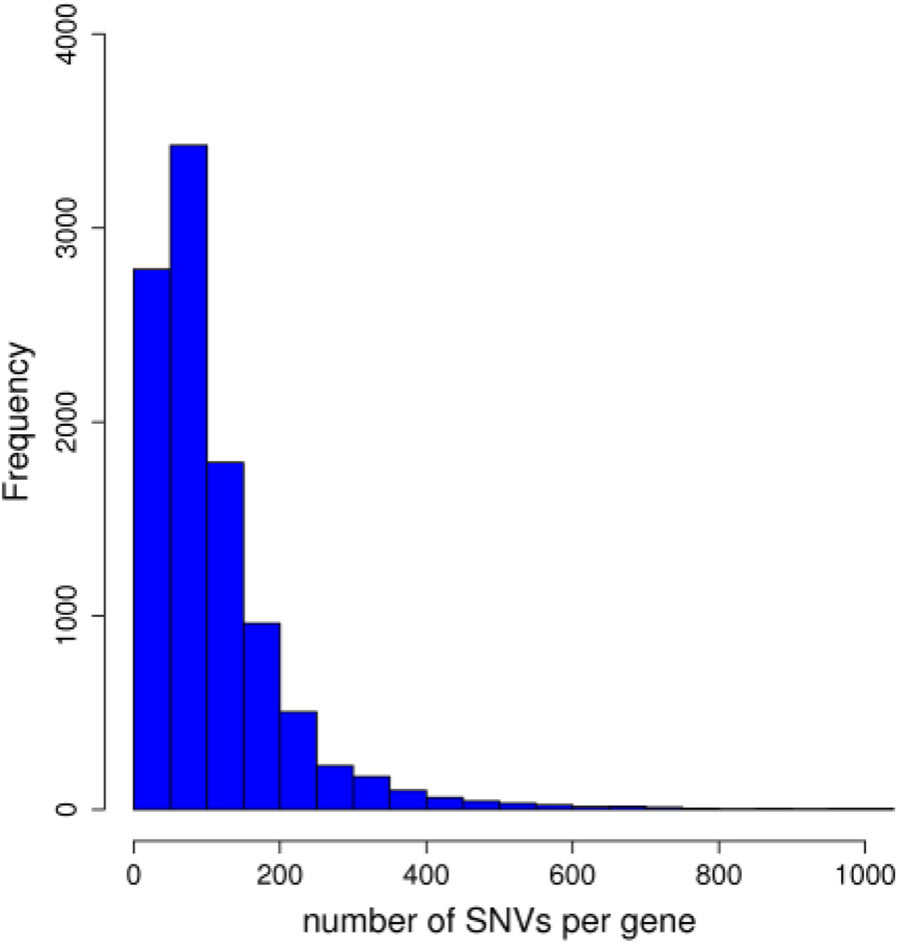
Number of SNVs with a MAF <5 % for each gene grouping

Both methods require specification of SNV sets for combined analysis. We restrict our attention to variants in exonic regions, and group SNVs by genes. We considered 8806 genes across the odd-numbered chromosomes. Of these, 135 contained only one SNV, while the obscurin protein-coding *(OBSCN)* gene on chromosome 1 had the maximum of 1054 SNVs in its exonic regions. Figure 1 shows the distribution of SNVs per gene.

**Fig. 2:**
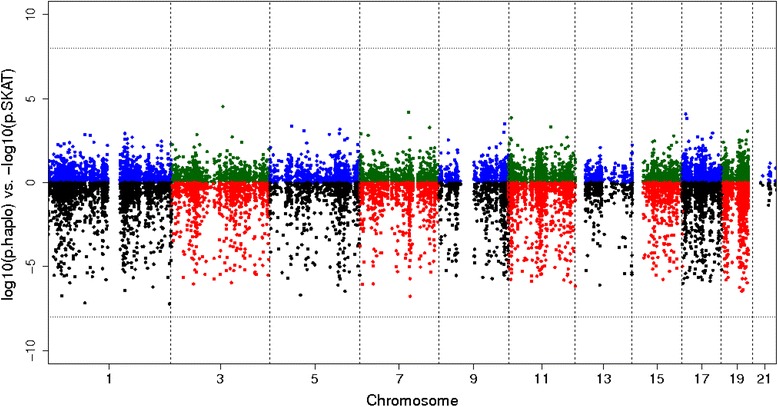
Association results for all odd chromosomes: the upper half represents SKAT -log10(*p* value); the lower half shows log10(*p* value) for the haplotype method

Type I error evaluation of the novel haplotype association method revealed an inflated type I error rate for genes with more than 14 haplotypes, but not for genes with fewer haplotypes (type I error rate = 0.053 at α = 0.05, 95 % confidence interval from 0.0495 to 0.0567). Hence, in Fig. 2 we report only results from genes with fewer than 14 haplotypes, after collapsing rare haplotypes together.

The most significant gene-based SKAT result was found in the activated leukocyte cell adhesion molecule (*ALCAM*) gene on chromosome 3; Table 1 lists the top ten SKAT association results. This gene codes for an immunoglobulin protein that is expressed in neural and epithelial cells [10]. The association *p* value with the set of 28 *ALCAM* SNVs was 7.0 × 10^−5^. However, none of the SKAT gene-based tests reached the genome-wide significance threshold, with Bonferroni correction for the 8806 genes tested (5.7 × 10^−6^).

**Table 1:** Top 10 signals from gene-based SKAT

Gene	Chromosome	Position	# SNVs	SKAT *p* value	Haplotype *p* value
*ALCAM*	3	105243142	28	7.00E-05	0.0095
*LMTK2*	7	97784102	84	0.000162	0.028
*ZBTB4*	17	7365289	58	0.000138	0.0036
*UBQLNL*	11	5536309	30	0.000214	4.1E-05
*WDR16*	17	9480017	30	0.000269	0.019
*KCNN4*	19	44273159	22	0.000281	–
*SLC19A1*	21	46934932	85	0.000386	0.025
*TRPM5*	11	2426196	159	0.00042	0.19
*PRRX2*	9	132481557	12	0.000434	1.0E-04
*NNT*	5	43609313	35	0.000618	0.031

Two of the most significant genes found to be associated with DBP by the haplotype method were fucose-1-phosphate guanylyltransferase (*FPGT*) on chromosome 1 and secreted protein, acidic, cysteine-rich (*SPARC*) on chromosome 5. Both genes (*p*_*FPGT*_ = 6.7 × 10^− 8^; *p*_*SPARC*_ = 3.3 × 10^− 7^) reached the genome-wide significance using Bonferoni correction. Table 2 lists the top ten haplotype association results.

**Table 2:** Top 10 signals for gene-based haplotype analysis

Gene	Chromosome	Position	# SNVs	# Haplotype	Haplotype *p* value	SKAT *p* value
*ADSS*	1	244572897	3	3	5.9E-08	0.43
*FPGT*	1	74670081	5	5	6.7E-08	1.4E-03
*MYL10*	7	101256771	4	6	1.7E-07	2.1E-03
*FGR*	1	27939439	2	3	1.8E-07	0.12
*KIF2A*	5	61642994	5	3	2.0E-07	0.91
*SPARC*	5	151043147	3	4	3.3E-07	8.5E-02
*CYP2A13*	19	41594384	8	6	3.4E-07	0.32
*PKLR*	1	155260382	3	4	3.7E-07	1.2E-03
*CADM4*	19	44127515	2	3	4.2E-07	4.7E-02
*ZNF529*	19	37037771	7	7	5.7E-07	0.22

## Discussion

The top gene association from the SKAT analysis, *ALCAM*, has been identified in a quantitative trait locus for systolic blood pressure in previous literature [11] and has shown differential gene expression in rats with hypertension [12].

The meta-analysis of haplotype results identified as its second strongest signal the gene *FPGT*, a gene in LD with a single nucleotide polymorphism (SNP) previously reported to be associated (*p* = 7.2 × 10^− 5^) with DBP in the lymphoblastoid cell line [13]. Our haplotype methods detected a much stronger association between *FPGT* and DBP (*p* = 6.7 × 10^− 8^). This stronger signal might be the result of the joint impact of all the five SNVs located in this gene. The sixth most significant gene identified by the haplotype approach*, SPARC*, contains three variants and was previously shown to be associated with cardiac dysfunction [14].

## Conclusions

We performed gene-based multi-SNV analyses to identify regions of the genome associated with DBP. While we observed some consistencies between the SKAT and haplotype analyses, the haplotype analysis revealed multiple genome-wide significant results, some in genes that have been previously implicated in blood pressure regulation. Further investigation in a larger number of participants is needed to confirm the novel associations identified in this report.
